# Adrenal Schwannoma: A Rare but Important Consideration in Adrenal Tumors

**DOI:** 10.7759/cureus.83282

**Published:** 2025-05-01

**Authors:** Tariq Abdul Hamid, Khawla Yaser, Khaldon Abo Alsel, Rafe Alhayek, Yaser Saeedi

**Affiliations:** 1 Surgery, Dubai Health, Dubai, ARE; 2 Urology, Dubai Health, Dubai, ARE

**Keywords:** abdominal mass, adrenal malignancy, adrenal mass, benign peripheral nerve sheath tumor, incidental adrenal schwannoma, retroperitoneal schwannoma, robotic adrenalectomy, s100-positive tumor

## Abstract

Adrenal schwannomas (AS) are rare and are often detected incidentally on imaging studies. Due to their nonspecific imaging characteristics and typically normal endocrine profiles, definitive diagnosis relies on histopathological examination. This case report presents a 72-year-old male patient with a 4.6 cm right adrenal mass, initially suspected to be an adrenal adenoma on ultrasound. A contrast-enhanced computed tomography (CT) scan later characterized the lesion as a well-defined, heterogeneous, loculated mass with an absolute washout of 40% and a relative washout of 18.2%, raising concerns about its malignant potential. Endocrine evaluation confirmed its nonfunctional nature. Given its increasing size and unclear nature, surgical excision via robot-assisted transperitoneal adrenalectomy was performed. Histopathological and immunohistochemical analyses confirmed the diagnosis of a benign adrenal schwannoma. This case highlights the diagnostic challenges and therapeutic approach to AS, emphasizing the role of surgical resection in managing adrenal incidentalomas with indeterminate malignant potential.

## Introduction

Schwannoma, a rare tumor originating from Schwann cells of the myelinated neural sheath [1], can arise from the phrenic nerve, vagus nerve, and sympathetic trunk, which innervate the adrenal glands. While schwannomas predominantly occur in the head, neck, and extremities [1], adrenal schwannomas (AS) are exceedingly rare, comprising only 1%-3% of adrenal tumors with approximately 80 cases reported globally [2,3]. Due to their rarity, preoperative diagnosis is challenging, as there are no pathognomonic imaging features [4], making histopathological and immunohistochemical analyses crucial for definitive diagnosis [3]. A thorough understanding of AS clinical presentation, diagnostic challenges, and management strategies remains essential [1]. This case report describes a 4.2 cm right AS in a male patient, diagnosed histopathologically and removed robotically due to concerns over malignancy and increasing tumor size.

## Case presentation

**Figure 1:**
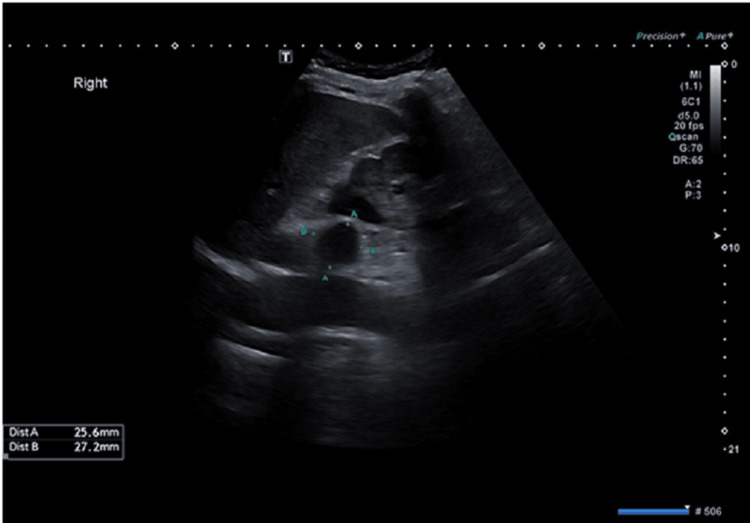
A 2.5 x 2.7 cm hypoechoic lesion in the right adrenal reported as suspicious of adrenal adenoma. However, the absence of definitive imaging characteristics warranted further evaluation with cross-sectional imaging.

A 72-year-old male patient with a medical history of type 2 diabetes mellitus and hypothyroidism, both managed on regular medications, presented to the urology clinic following the incidental detection of a 2.7 × 2.5 cm hypoechoic nodule on ultrasound. The lesion was initially suspected to be an adrenal adenoma (Figure 1). The patient was asymptomatic, with no history of hypertension, electrolyte imbalances, palpitations, weight loss, or Cushingoid features such as easy bruising or proximal muscle weakness, nor any abdominal symptoms.

**Figure 2:**
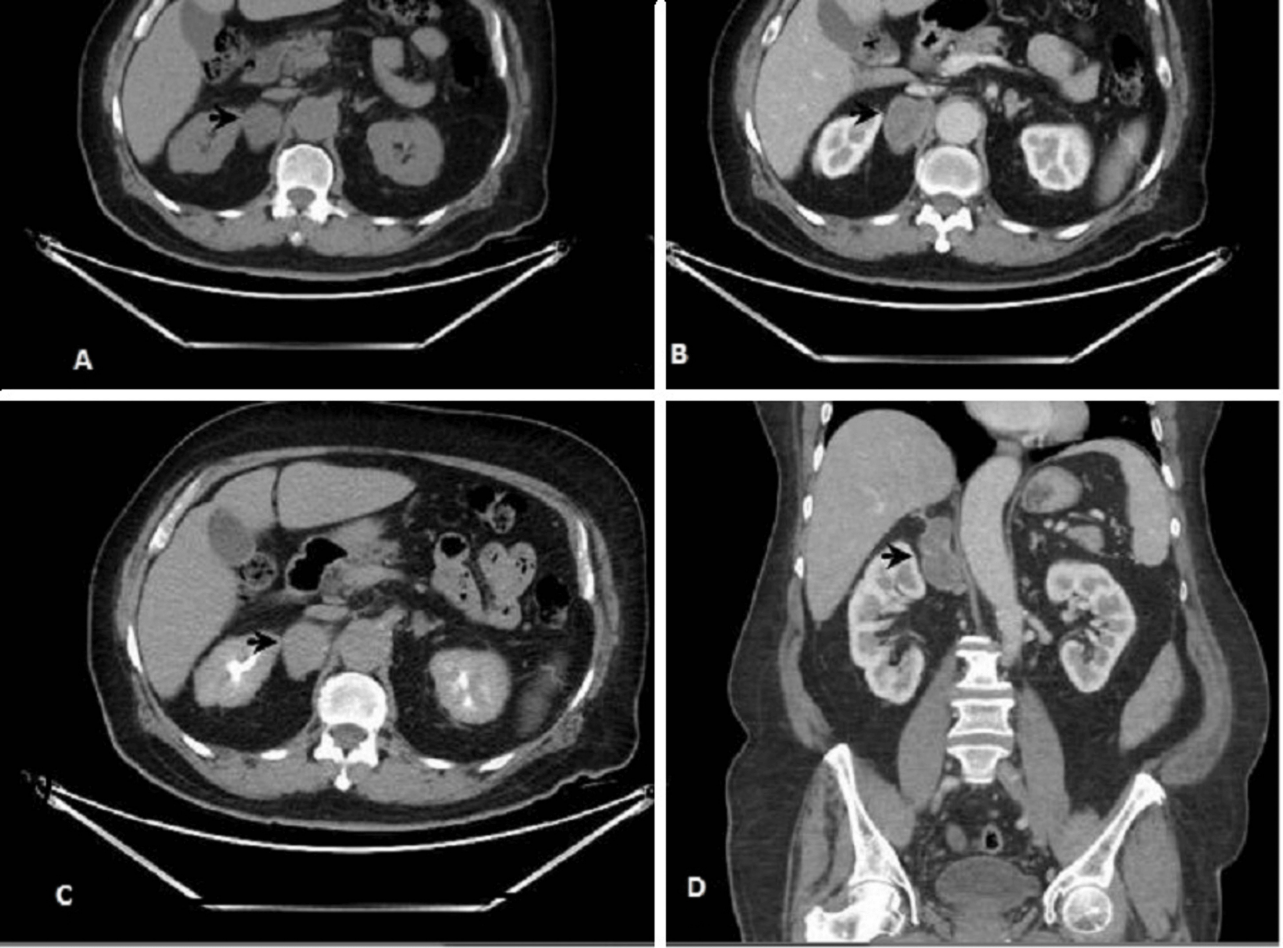
CT with contrast study. (A) Precontrast phase; mass in the right adrenal gland, 30 HU in noncontrast image; (B and D) mass enhancing to 55 HU in the portal venous phase. The mass is clearly localized to the right adrenal region, separate from the kidney. No significant invasion of adjacent structures, supporting its benign nature. (C) Persistent enhancement of the lesion to 65 HU in the delayed phase. The lesion exhibits mild progressive enhancement, a characteristic feature of schwannomas due to their fibrous nature. CT: computed tomography

Upon referral to the endocrinology service, a complete endocrine evaluation was performed to assess the functional status of the adrenal lesion. Hormonal investigations, including serum cortisol, aldosterone-renin ratio, and catecholamine metabolites, confirmed the mass was nonfunctional (Table 1). Eight weeks later, a follow-up contrast-enhanced computed tomography (CT) scan demonstrated a well-defined, heterogeneous, loculated mass in the right suprarenal region adjacent to the adrenal gland, measuring 4.2 × 3.5 × 6 cm. The lesion exhibited an absolute washout of 40% and a relative washout of 18.2%, raising concerns regarding its potential malignancy (Figure 2). Given its increasing size and the inability to exclude malignancy, the case was discussed in a multidisciplinary tumor board meeting, where surgical intervention was recommended.

**Table 1:** Hormonal workup of the index patient indicating a nonfunctioning adrenal mass. TSH: thyroid-stimulating hormone

Hormonal workup	Results	Reference range
Cortisol, morning	25	133-537 nmol/L
Aldosterone, upright	9.63	2.52-39.2 ng/dL
Renin, direct upright	3.67	2.8-39.9 mIU/mL
Aldo.Renin ratio upright	2.62	<2.7 ng/dL/mIU/mL
Metanephrine	<50	<70 ng/L
Normetanephrine	74	<120 ng/L
3-Methoxytyramine	<18	<15 ng/L
Free T4	17.5	12-22.0 pmol/L
TSH	2.790	0.27-4.2 mIU/mL

**Figure 3:**
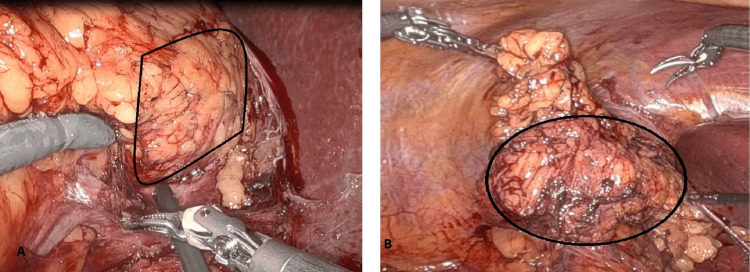
(A) Intraoperative images captured the robotic approach to adrenalectomy identifying the right adrenal schwannoma, highlighting meticulous dissection of the lesion from adjacent structures while preserving critical vascular anatomy. (B) The minimally invasive approach facilitated precise tumor resection with minimal blood loss.

**Figure 4:**
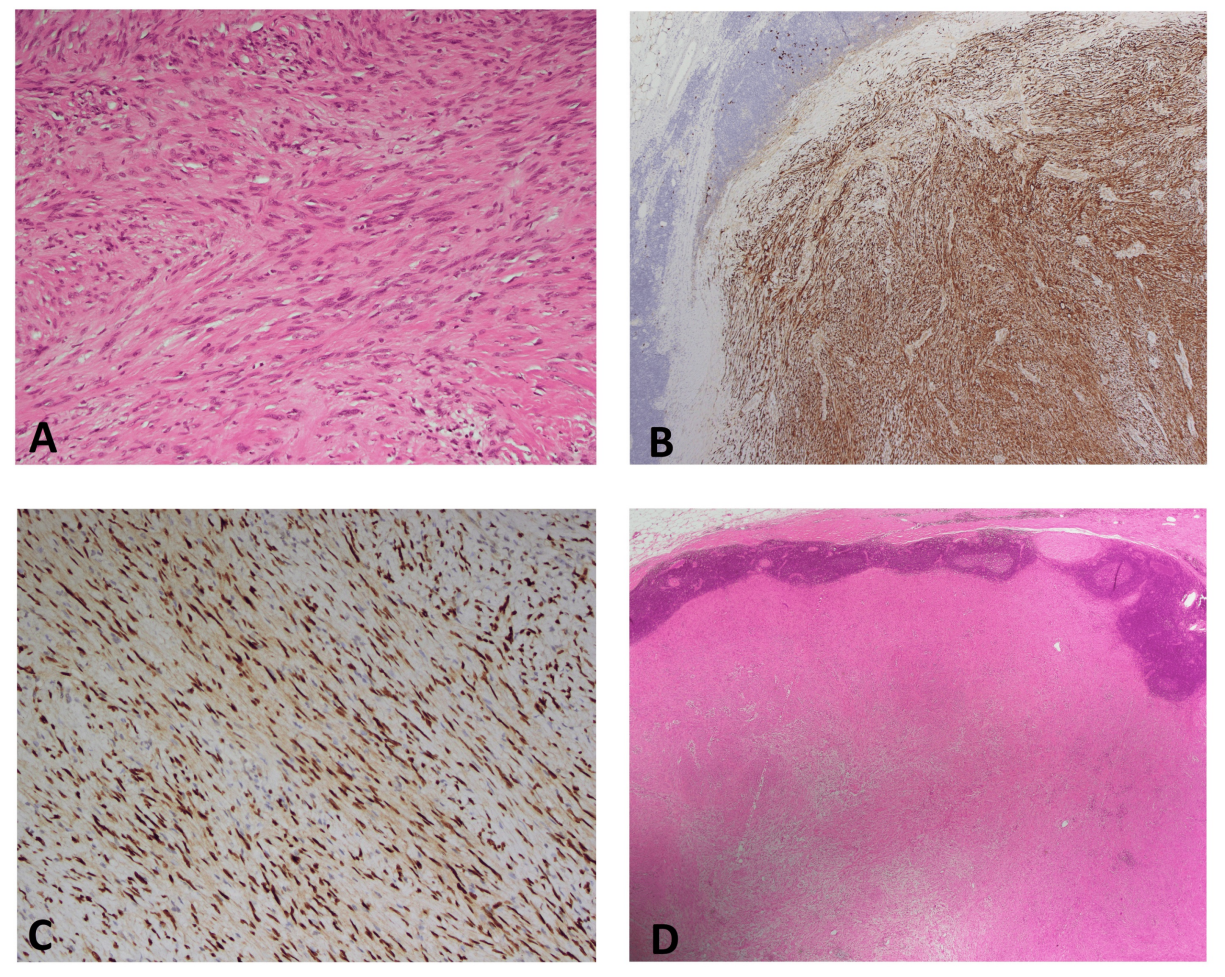
(A) Spindle cell tumor lesion composed of spindled stromal cells with fibro-collagenous stroma, H&E x200. (B) Tumor nodular spindle cell strongly and diffusely positive for S100 protein, x40. (C) Tumor spindle cells are positive for SOX10 strong nuclear staining of tumor spindle cells SOX10, x200. (D) Well-defined capsulated spindle cell tumor nodule rimmed by nodal lymphoid tissue with reactive lymphoid follicles and extranodal adipose tissue, H&E x20.

The patient subsequently underwent a robotic-assisted transperitoneal right adrenalectomy using a four-port technique. Intraoperatively, the mass was found to be encapsulated, adherent to the adrenal gland but without evidence of local invasion. The procedure was uneventful, with minimal intraoperative blood loss. The patient was discharged on postoperative day three in stable condition with no complications (Figure 3). Gross examination of the excised specimen revealed a well-circumscribed, solid, homogenous yellow-tan to gray-white firm nodular mass measuring 4.9 × 2.6 × 2.9 cm and weighing 80 g. Microscopic evaluation identified a spindle cell tumor composed of compact Antoni A areas with nuclear palisading and loosely arranged Antoni B areas (Figure 4A). Immunohistochemical staining was diffusely positive for S100 and SOX10 (Figures 4B, 4C), confirming the diagnosis of a benign peripheral nerve sheath tumor consistent with AS. The tumor was well-encapsulated and surrounded by lymphoid tissue with reactive lymphoid follicles and extranodal adipose tissue (Figure 4D). The patient had an uneventful recovery and was followed up two months postoperatively. He remained asymptomatic with no evidence of recurrence or residual disease. Long-term follow-up with annual imaging and endocrine assessment was recommended to monitor for potential recurrence or new adrenal lesions.

Contrast-enhanced CT imaging showed a well-defined, heterogeneous mass in the right suprarenal region measuring 4.2 x 3.5 x 6 cm. The lesion exhibited mild enhancement with an absolute washout of 40% and a relative washout of 18.2%, findings that are not definitive for either benign or malignant adrenal tumors, necessitating histopathological confirmation.

## Discussion

Schwannomas, also known as neurilemmomas [5], originate from Schwann cells that envelop peripheral nerves, except for cranial nerves I and II, which lack these cells [5]. Although schwannomas are predominantly located in the head, neck, and flexor surfaces of the extremities [1], retroperitoneal involvement is uncommon (approximately 5%), with AS being a rare subset [3-6]. AS accounts for only 0.2%-0.5% of adrenal tumors [2]. In a study by Goh et al., seven retroperitoneal schwannomas were reported, four of which were suspected to be adrenal in origin [7]. These tumors were first described by Verocay in 1908 [5] and are largely benign, with unknown etiology in nearly 90% of cases [4]. However, they may occasionally occur in association with syndromes such as neurofibromatosis type 2, Carney complex, and familial schwannomatosis [8]. Malignant transformation is rare but more commonly linked with neurofibromatosis type 1 or 2 [9].

AS typically present in middle-aged individuals, with a slight female predominance, and are frequently detected incidentally on imaging [2]. A recent study analyzing 33 cases of AS reported an age range of 14-89 years, a median tumor size of 5.5 cm, and a predominance in female patients [3-6]. These tumors are generally solitary, well-defined, and spherical [5]. Most patients remain asymptomatic due to the nonfunctional nature of the tumor [1], though larger lesions (>4 cm) may cause nonspecific abdominal, flank, or back pain [2]. In a case series of 31 patients, 84% of AS were incidental findings [9]. Reported tumor sizes range from 0.6 to 14.5 cm, with a median of 5.5 cm [1]. Larger tumors (>8-10 cm) frequently exhibit degenerative changes, including cystic areas, calcifications, interstitial fibrosis, and hyalinization [5], further complicating imaging-based diagnosis [1]. CT and magnetic resonance imaging (MRI) are valuable tools for evaluating adrenal lesions; however, imaging alone cannot reliably differentiate schwannomas from malignant tumors due to their encapsulated, hypervascular, and heterogeneous nature [3-6]. Nonetheless, imaging remains crucial for assessing tumor size, location, and potential invasion of adjacent structures, aiding surgical planning [5].

Routine endocrine evaluation of incidental adrenal tumors is recommended before surgery to determine functional status and guide treatment decisions [1]. While some reports suggest that AS may be associated with excessive corticosteroid or adrenomedullary hormone secretion [1], most cases, including the present one, show no biochemical abnormalities [9]. Histopathological confirmation is essential, revealing two distinct patterns: Antoni A (cellular areas with nuclear palisading) and Antoni B (paucicellular areas) [9]. Immunohistochemical staining is diagnostic, with tumor cells expressing S100 protein and vimentin, differentiating schwannomas from other pathologies [10]. Additional staining for calretinin, as demonstrated by Fine et al., aids in confirming the diagnosis [11].

Management of adrenal incidentalomas depends on functional status, tumor size, and malignancy risk [1]. The 2002 National Institutes of Health guidelines recommend resection for lesions >6 cm, conservative management for those <4 cm with benign imaging features, and individualized decision-making for tumors between 4 and 6 cm [6]. Literature suggests that tumors >4 cm have an increased malignancy risk (>25% for lesions >6 cm), warranting surgical intervention, as in this case [1]. Resection is also indicated for tumors demonstrating rapid growth, irregular margins, necrosis, or invasion of adjacent structures [1].

Despite the diagnostic challenges, primary AS have an excellent postsurgical prognosis. A systematic review of 85 cases showed no recurrence or metastasis over a median follow-up of 45 months [2]. Our patient remains asymptomatic two months postadrenalectomy, reinforcing the favorable outcome associated with the surgical management of AS. Given the rarity of AS and the lack of definitive nonhistologic diagnostic modalities, it should be considered in the differential diagnosis of incidentally discovered adrenal masses [5].

## Conclusions

AS are rare, often incidentally detected lesions that pose significant diagnostic challenges due to their imaging resemblance to malignant adrenal tumors. This case underscores the importance of a multimodal diagnostic approach, incorporating imaging, endocrine evaluation, and ultimately histopathological confirmation. Given the potential for growth and the uncertainty in preoperative characterization, surgical excision remains a prudent strategy, particularly for nonfunctional adrenal tumors exceeding 4 cm. This case reinforces the role of robotic-assisted adrenalectomy as an effective minimally invasive technique for managing indeterminate adrenal masses, ensuring complete tumor removal with favorable postoperative outcomes. Awareness of AS among urologists, endocrinologists, and radiologists is essential to optimize patient management and improve diagnostic accuracy.
